# Phosphatases in toll-like receptors signaling: the unfairly-forgotten

**DOI:** 10.1186/s12964-020-00693-9

**Published:** 2021-01-25

**Authors:** Valérie Lannoy, Anthony Côté-Biron, Claude Asselin, Nathalie Rivard

**Affiliations:** Department of Immunology and Cell Biology, Cancer Research Pavilion, Faculty of Medicine and Health Sciences, Université de Sherbrooke, 3201, rue Jean Mignault, Sherbrooke, QC J1E4K8 Canada

**Keywords:** Toll-like receptors, TLRs, Phosphatases, NF-κB, IRFs axis, IFN I, MAPK

## Abstract

**Abstract:**

Over the past 2 decades, pattern recognition receptors (PRRs) have been shown to be on the front line of many illnesses such as autoimmune, inflammatory, and neurodegenerative diseases as well as allergies and cancer. Among PRRs, toll-like receptors (TLRs) are the most studied family. Dissecting TLRs signaling turned out to be advantageous to elaborate efficient treatments to cure autoimmune and chronic inflammatory disorders. However, a broad understanding of TLR effectors is required to propose a better range of cures. In addition to kinases and E3 ubiquitin ligases, phosphatases emerge as important regulators of TLRs signaling mediated by NF-κB, type I interferons (IFN I) and Mitogen-Activated Protein Kinases signaling pathways. Here, we review recent knowledge on TLRs signaling modulation by different classes and subclasses of phosphatases. Thus, it becomes more and more evident that phosphatases could represent novel therapeutic targets to control pathogenic TLRs signaling.

**Graphic abstract:**

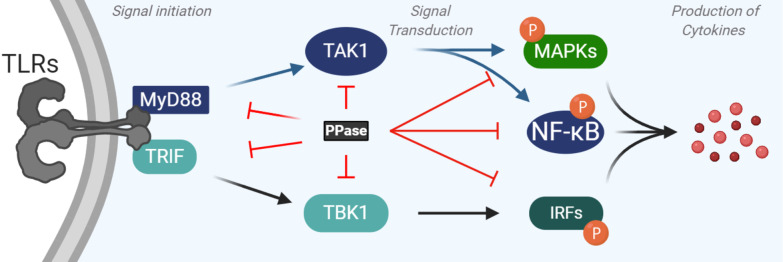

**Video Abstract**

## Background

Toll-Like Receptors (TLRs) belong to the Pattern Recognition Receptors (PRRs) superfamily that recognizes Microbe-Associated Molecular Patterns (MAMPs)—not necessarily found on pathogens—, PAMPs (Pathogen) and DAMPs (Damage) [1]. There are 13 identified TLRs in mammals playing an instrumental role in the regulation of the innate immune system [2]. These receptors are highly conserved proteins also found in the phylum Cnidaria, including jellyfishes [3]. As TLRs evolved before the adaptive immune system, they constitute an indispensable first line of defense [4]. Accordingly, TLRs expression is not restricted to immune cell lineages. *TLR3* and *TLR5* mRNAs are detected ubiquitously within the human body, while other TLRs are mostly expressed in epithelial tissues (except TLR10, more restricted) [5]. Thus, because they constitute the first line of defense against microbes (see below), TLRs are involved in pathogenesis of various disorders. Several Single Nucleotide Polymorphisms (SNPs) of TLRs, their co-receptors and some adaptors are indeed associated with several diseases including infections, atherosclerosis, asthma, chronic cardiomyopathy and colorectal cancer [6, 7].

TLRs are divided into two groups: those localized at the plasma membrane and those localized at the endosomal membrane [8]. Plasma membrane-localized TLRs recognize conserved motifs on extracellular microorganisms such as bacteria, fungi, protozoan and helminth parasites [9]. Yet, plasma membrane TLRs are also able to bind DAMPs. The most glaring example is TLR4 that is well-known to recognize Lipopolysaccharide (LPS), a component of Gram-negative bacteria, and 28 other ligands including DAMPs [10]. This wide range of recognition is allowed by combinations between TLRs homodimers or heterodimers with co-receptors and/or accessory molecules [11]. The other plasma membrane TLRs include TLR1, TLR2 and TLR6 which bind various lipopeptides from Gram-positive bacteria and TLR5 which binds flagellin from flagellated bacteria [9].

Endosomal TLRs (TLR3, TLR7, TLR8, TLR9) are activated by nucleic acids derived from microbes. Their ectodomains undergo proteolytic cleavage in endosomes to generate functional receptors for nucleic acid structures [12]. For instance, TLR3 recognizes double stranded RNA (dsRNA), TLR7 and TLR8 recognize single stranded RNA (ssRNA) and TLR9 binds unmethylated cytidine-phosphate-guanosine (CpG) dinucleotides [13].

Notably, half of the TLRs exhibit both plasma membrane and endosomal localizations. For instance, in intestinal epithelial cells exposed to bacterial DNA, TLR9 moves from the endosomal compartment to the cytoplasmic membrane [14]. In macrophages, membrane-associated TLR4 reaches the endosomal compartment through endocytosis [15]. A similar process has been described for TLR2 which is endocytosed after its heterodimerization with TLR1 or TLR6 in myeloid cells [16].

Binding of ligands to TLRs triggers the activation of several intracellular signaling pathways involving protein phosphorylation, dephosphorylation and ubiquitination. While the roles of specific protein kinases in this process have been well described [17, 18], much less is known about the implications of phosphatases, particularly tyrosine phosphatases. In this review, we summarize how specific phosphatases regulate TLRs signaling and function.

## TLR signaling pathways: an overview

### Rapid tyrosine phosphorylation following TLRs engagement

TLRs are type I membrane glycoproteins with an ectodomain consisting of Leucine-Rich Repeats (LRRs), responsible for the molecular recognition of ligands [19]. TLRs contain also a cytoplasmic domain called Toll/Interleukin-1 Receptor (TIR) domain, which recruits several adapter proteins including Myeloid Differentiation primary response 88 (MyD88), Toll/Interleukin-1 Receptor domain-containing Adapter Protein (TIRAP) or TIR-domain-containing adapter-inducing Interferon-β (TRIF). Upon ligand binding, TLRs undergo conformational changes inducing homodimerization or heterodimerization which unveil TIR domains which can now recruit and bind different downstream signaling effectors regulating the host inflammatory response [20].

**Fig. 1:**
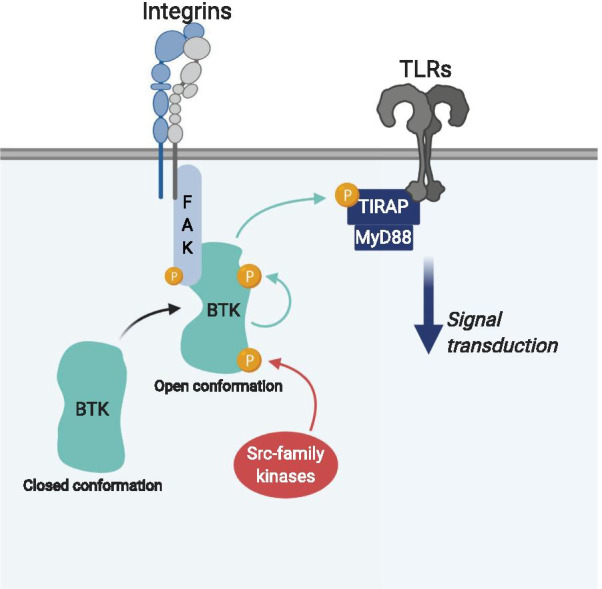
Tyrosine phosphorylation following TLRs engagement. Activated FAK phosphorylates BTK, opening its conformation and allowing its phosphorylation and activation by Src kinases [29, 30]. BTK can then phosphorylate adaptor proteins such as TIRAP

Notably, the TIR domain of TLR2, 3, 4, 5, 8 and 9 is rapidly phosphorylated on tyrosine upon stimulation with their respective ligands. This phosphorylation is required for the recruitment of adapter proteins and subsequent activation of the downstream signaling cascades [17, 21, 22]. Several tyrosine kinases interact with TLRs, including Src, Bruton’s Tyrosine Kinase (BTK), Lyn and Syk. The Focal Adhesion Kinase (FAK) is also rapidly activated upon ligand binding to TLR. Indeed, FAK phosphorylation is observed in macrophages isolated from mice deficient for MyD88, one of the first adapter molecule recruited following TLR4 activation (see below), suggesting that FAK activation occurs early after TLR activation [23]. FAK is indeed required for TLR4 downstream signaling since cytokine induction in response to LPS is totally abrogated in *Fak*^−/−^ cells. However, FAK does not possess TIR domains for direct interaction with MyD88 or TLR4 [24]. Instead, activated FAK phosphorylates BTK which interacts with the TIR domains of TLR4 (but also of TLR6, 8, and 9) [25]. BTK phosphorylation by FAK opens its conformation [26] allowing its phosphorylation and activation by Src kinases (Fig. 1). Once activated, BTK phosphorylates different adapter molecules such as the bridging adapter TIRAP, facilitating Myd88 recruitment to TLR4 [27]. Interestingly, BTK can also phosphorylate TLR3 and this is required for downstream signaling [28]. However, BTK does not phosphorylate TRIF, but rather facilitates TRIF interaction with effectors Since BTK expression is restricted to myeloid cells, ETK, a member of the TEC family of tyrosine kinases, is suspected to play similar functions in other cell types such as epithelial cells.

Aside from these observations, tyrosine phosphorylation of TLR3 and TLR4 also increased in response to EGF (Epidermal Growth Factor), and inhibition of EGFR (EGF Receptor) kinase activity impaired activation of their downstream signaling. In fact, the Src family kinases mediated this crosstalk between EGFR and TLRs, triggering the recruitment of adapters and other effector proteins [31, 32].

Altogether, these observations strongly suggest that a coordinated interplay should exist between tyrosine kinases and phosphatases in order to tightly regulate the activation status of TLRs intracellular signaling and cellular function. Below, we summarize the main signaling pathways activated by TLRs and we discuss how these pathways are modulated by various tyrosine and serine/threonine phosphatases.

### The MyD88-dependent signaling

**Fig. 2:**
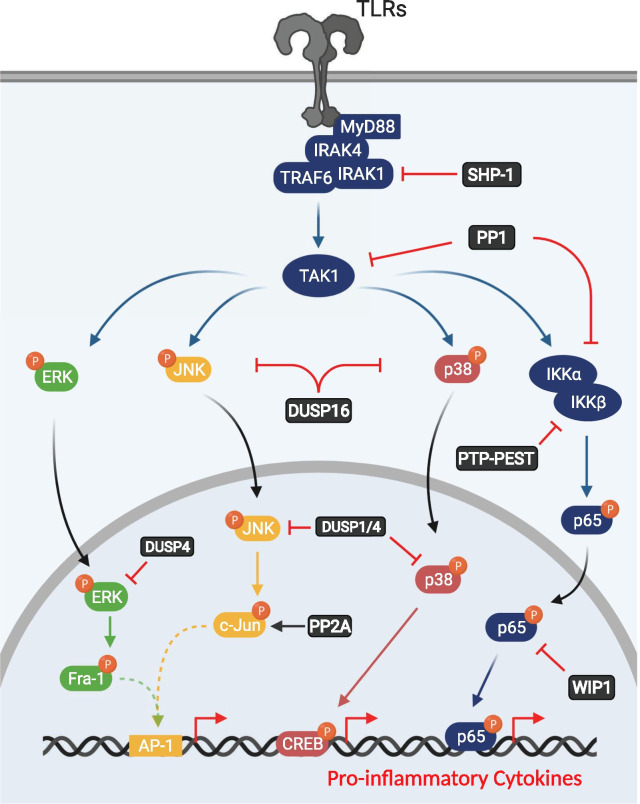
Regulation of TLR/MyD88-dependent pathways by phosphatases. TLRs localized at the plasma membrane use the bridging adapter TIRAP to recruit MyD88 and activate the NF-κB and MAPK pathways. MyD88 then recruits IRAK4 which phosphorylates IRAK1 which activates TRAF6. TRAF6 induces K63-linked polyubiquitination of TRAF6 itself serving as a platform leading to TAK1 autophosphorylation and activation. Activated TAK1 phosphorylates IKK-α and IKK-β which induce IκBα degradation, allowing NF-κB nuclear translocation and induction of proinflammatory genes transcription. TAK1 also phosphorylates the MAPK Kinases MKK3, MKK4, MKK6 and MKK7, resulting in activation and nuclear translocation of MAP Kinases which phosphorylate several transcription factors inducing proinflammatory mediator genes transcription

TLRs activate two types of pathways to control inflammatory responses: the MyD88-dependent pathway activated by all TLRs except TLR3, and the MyD88-independent but TRIF-dependent pathway activated directly by TLR3 and indirectly by other TLRs [33, 34]. Importantly, Myd88 contains a TIR domain as observed in most TLRs, but most TLRs use the bridging adapter TIRAP to recruit MyD88 [35]. Indeed, TIRAP contains a TIR domain at its C-terminus and a phosphatidylinositol-4,5 biphosphate (PIP_2_) binding motif at its N-terminus, required for recruitment to the plasma membrane [36]. Of note, TIRAP tyrosine phosphorylation by BTK is necessary for Myd88 recruitment to the plasma membrane (Fig. 1). Furthermore, *Tirap*-deficient mice revealed that TIRAP is crucial for the activation of MyD88-dependent signaling following TLR2 and TLR4 activation [37]. The N-terminus Death Domain (DD) of MyD88 recruits the Interleukin-1 Receptor-Associated Kinase 4 (IRAK4) via DD-DD domains interaction. Consequently, mice lacking *Irak-4* display an almost total irresponsiveness to LPS challenge [38]. The IRAK1/Toll-Interacting Protein (TOLLIP) complex in which TOLLIP acts as a TLR signaling inhibitor [39], is also recruited. Hyperphosphorylation of IRAK1 by IRAK4 dissociates TOLLIP and IRAK1, and IRAK1 can then recruit and activate the TNF Receptor-Associated Factor 6 (TRAF6) ubiquitin E3 ligase. TRAF6 promotes lysine (K)63-linked polyubiquitination of IRAK1, IKK-γ and TRAF6 itself. The K63-linked ubiquitin chains serve as docking sites for adapters TGF-β-Activated kinase 1-Binding 2 and 3 (TAB2, TAB3) which sequester TGFβ-Activated Kinase 1 (TAK1). Then, TAK1 auto-phosphorylates and becomes activated [40]. Hence, TAK1 activation mostly depends on TRAF6 E3 ubiquitin ligase [41]. Activated TAK1 then phosphorylates substrates such as IKKs (IκB-α kinases) and the MAPK Kinases (MAPKKs) MKK3, MKK4, MMK6 and MKK7 (Fig. 2) [42].

Activation of the IKK complex (IKK-α, IKK-β and IKK-γ) leads to NF-κB inhibitory fragment (IκB-α) degradation. This exposes the NF-κB nuclear localization sequence and allows NF-κB translocation to the nucleus to initiate transcription of proinflammatory genes, including those encoding chemokines and cytokines. Besides, stimulation of the MAPKKs results in activation and nuclear translocation of the MAPKs ERK1/2 (Extracellular signal-Regulated Kinases 1/2), p38 and c-Jun N-terminal kinases (JNK), which phosphorylate several transcription factors (such as AP-1), also inducing proinflammatory mediator genes transcription (Fig. 2).

### The TRIF-dependent signaling

**Fig. 3:**
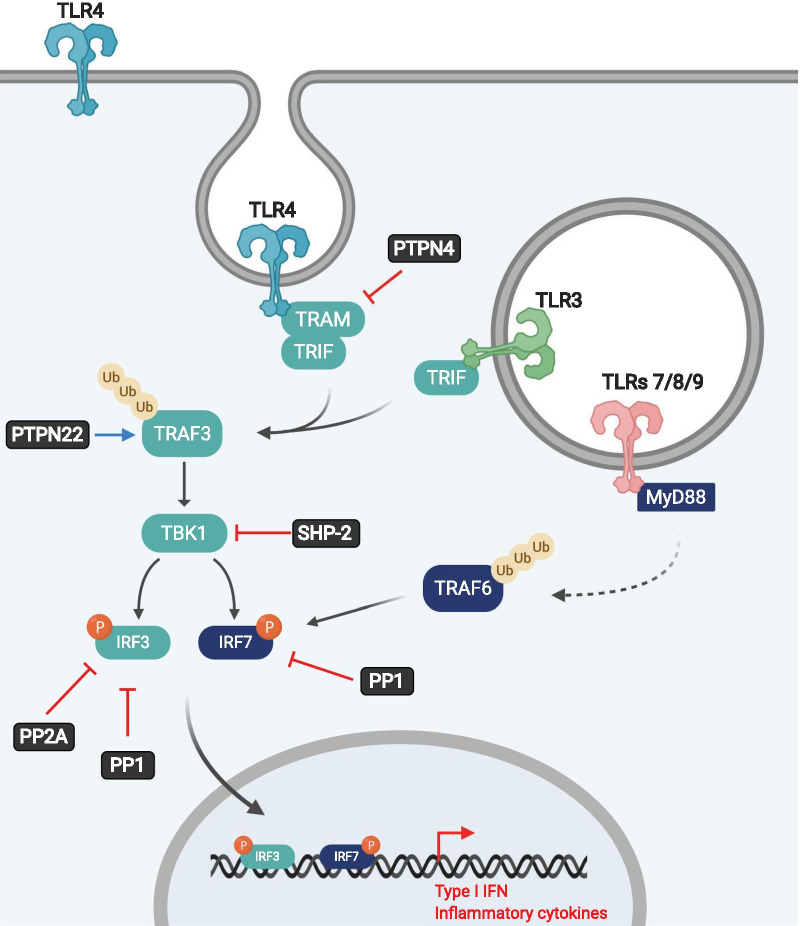
Regulation of TLR/TRIF-dependent pathways by phosphatases. TRIF is recruited directly to TLR3 but indirectly, via TRAM adaptor, to TLR4, following stimulation. TRIF adaptor then recruits TRAF3 which induces K63-linked ubiquitination of TBK1, its autophosphorylation and then, activation. Activated TBK1 then phosphorylates the transcription factors IRF3 and IRF7, leading to homodimerization or heterodimerization, translocation into the nucleus and target genes induction, including *IFNI*. TLR7, TLR8 and TLR9 activate the TRIF-dependent pathways via the MyD88 adaptor

TRIF is an adapter protein upstream of the production of type I interferons (IFN-α and IFN-β) and other proinflammatory mediators. TRIF is recruited directly to TLR3 but indirectly to TLR4 via the TRIF-Related Adapter Molecule (TRAM) [43]. In fact, TLR4 initially recruits TIRAP and MyD88 at the plasma membrane and is subsequently endocytosed to the endosomes where it recruits TRAM and TRIF. TRAM mediates the activation of TRIF which associates with TRAF3 and TRAF6. The TRAF6 complex then induces RIP-1 (Receptor-Interacting serine/threonine-Protein kinase-1) which activates the IKK-α, β, γ complex and then NF-κB [44, 45]. Furthermore, TRAF3 triggers the K63-linked ubiquitination of TANK-Binding Kinase 1 (TBK1) (47)] and IKK-ε (48)]. K63-linked ubiquitination chains act as scaffolds for kinases, inducing their catalytic activation [46–48]. Activated TBK1 then phosphorylates the transcription factor family IRFs (Interferon Regulatory Factors) [49], leading to homodimerization or heterodimerization, translocation into the nucleus and target genes expression, including *IFNI* (encoding IFN-α and IFN-β) [50] (Fig. 3).

Likewise, TLR3 signaling is initiated from endosomal membranes where it activates TRAF6 and TRAF3. Then, TRIF signals through TRAF3, TBK1, and IKK-ε to initiate IRF-mediated transcription. TRAF3 therefore acts as a critical component to trigger TRIF-dependent signaling pathways (Fig. 3). This is exemplified by the phenotype of *Traf3*-deficient myeloid and dendritic cells in which IFN production is impaired upon TLR4, TLR7 and TLR9 stimulations [51, 52]. Interestingly, analysis of gene expression profiles in dendritic cells carrying a loss of function mutation in *Trif* reveals that 47% of LPS-responsive genes are TRIF-dependent [53].

## The human protein phosphatome

The human protein phosphatome comprises a set of genes encoding phosphatases that remove phosphate groups from proteins. Protein phosphatases were first classified according to their catalytic domain annotation [54]. Today, phosphatase families are subdivided into classes, according to their preferred substrates [55]. A simplified classification is presented in Table 1.

**Table 1:** The human protein phosphatome classification

	Members	Properties	Targets	Members	References
**PTPs**	108	Need catalytic C-oxidation	pY	SHP-1, Cdc25b, etc	[56, 57]
Classical PTPs	41	Dephosphorylation of various proteins	pY	RTPs, PTP1B	[58, 59]
DSPs DUSPs	63	Dephosphorylate MAPKs	pY/pS/pT	PTEN, MKPs	[60, 61]
LMW-PTP	1	Dephosphorylates growth factor receptors	pY/pT	LMW-PTP	[62, 63]
Cdc25	3	Dephosphorylate cyclin-dependent kinases	pY/pT	Cdc25a, Cdc25c	[64, 65]
**PPPs**	13	No metal ion or Ca^2+^-dependent	pS/pT	PP1, PP2A, PP2B	[66]
**PPMs**	15	Require Mn^2+^/Mg^2+^ ions	pS/pT	PP2C/PDH, WIP1	[67, 68]
**HADs**	17	Need catalytic D-oxidation	pS/pY	Eya, FCP1, SCP	[69, 70]

In bold: protein phosphatases families

C, Cysteine; Cdc25, Cell division cycle 25; D, Aspartate; DSPs/DUSPs, Dual-Specificity Phosphatases; FCP1, F-Cell Production 1; HADs, Holo-Acid Dehalogenases; LMW-PTP, Low Molecular Weight-Protein Tyrosine Phosphatases; MKPs, MAPK Phosphatases; PDH, Pyruvate Dehydrogenase Phosphatase; PP1, Protein Phosphatase 1; PP2A, Protein Phosphatase 2 A; PP2B, Protein Phosphatase 2 B; PP2C, Protein Phosphatase 2 C; PPMs, Metal-dependent Protein Phosphatase; PPPs, Phospho-Protein Phosphatases; pS, Phosphoserine; pT, Phospho-threonine; PTEN, Phosphatase and TENsin homolog; PTPs, Protein Tyrosine Phosphatases; PTP1B, Protein Tyrosine Phosphatase 1B; pY, Phospho-tyrosine; RTPs, Receptor Tyrosine Phosphatases; SCP, Small Carboxy-terminal domain Phosphatase; SHP-1, Src Homology region 2 domain-containing Phosphatase-1; WIP1, Wild-type p53-Induced Phosphatase 1

### Classical protein tyrosine phosphatases (PTPs)

#### Protein tyrosine phosphatase 1B (PTP1B)

PTP1B is a cytoplasmic protein tyrosine phosphatase expressed in many tissues [71, 72] and which targets a wide range of substrates [73–75]. Notably, *Ptp1b* gene knockdown in mouse RAW264.7 macrophages results in increased NF-κB and IRFs activation in response to TLR3, TLR4 and TLR9 stimulation [76]. Similarly, macrophages derived from *Ptp1b*^*−/−*^ mice exhibit accelerated IκBα degradation following TLR4 activation by LPS [77]. Elevated concentrations of inflammatory cytokines and IFN I were also found in the lungs of *Ptp1b*^*−/−*^ mice following intranasal administration of Oligo-Deoxy-Nucleotide 1826 (ODN 1826, a TLR9 agonist) [78]. Thus, these studies suggest that PTP1B restrains TLR4 and TLR9-induced inflammatory responses. Conversely, PTP1B expression levels were significantly increased in the brain 24 h after LPS injection in mice. In addition, when overexpressed in microglial cells, PTP1B increased LPS-induced TNF-α (Tumor Necrosis Factor-α), iNOS (inducible Nitric Oxide Synthase) and IL-6 (Interleukin 6) expression as well as NO production. It has been demonstrated that PTP1B exerts such pro-inflammatory function by dephosphorylating a negative regulatory site (tyrosine 527) on Src, hence activating its kinase activity and downstream NF-κB signaling [79, 80]. These studies suggest cell type-specific roles for PTP1B in TLRs signaling.

#### Protein tyrosine phosphatase non-receptor type 4 (PTPN4)

PTPN4 tyrosine phosphatase regulates T Cell Receptor signaling [81]. Huai et al*.* have reported that PTPN4 dephosphorylates TRAM adapter, hence inhibiting TRIF-dependent TLR4 signaling [82]. Additionally, *Ptpn4* silencing in mouse macrophages increases IRF3 phosphorylation in response to LPS but not in response to Poly I:C, a TLR3 ligand. Ectopic expression of a catalytically inactive mutant of PTPN4 in RAW264.7 macrophages abolishes LPS-induced IFN-β secretion [82]. Biochemical and genetic analyses demonstrate that PTPN4 dephosphorylates TRAM adapter on tyrosine 167 – within the TIR domain –, thereby disrupting TRAM-TRIF direct interaction [82] (Table 2). Thus, PTPN4 is a negative regulator of TLR4/TRIF-dependent signal transduction.

**Table 2:** Protein phosphatases involved in TLRs signaling

	Targets	Residues	Upstream	Effects	Cell types	References
PTPN4	TRAM	Y167	TLR4		Peritoneal and RAW264.7 macrophages	[82]
PTP-PEST	IKK-β	Y188 Y199	TLR3, TLR4, TLR9	↓	RAW264.7 macrophages	[104]
DUSPs (1, 4, 16)	ERK1/2	T202 Y204	TLR2, TLR4, TLR9	↓	RAW264.7 macrophages and BMMs	[105–110]
	JNK1/2	T183/Y185				
	P38	T180/Y182				
PP1	IKK-α	S180	TLR4	↓	RAW264.7 macrophages	[111]
	IKK-β	S181				
	TAK1	S412	TLR3, TLR4, TLR9			[112]
	IRF3	S385 S396	TLR3, TLR4		RAW264.7 macrophages and BMMs	[113]
	IRF7	S471 S472 S477 S479	TLR3		RAW264.7, U-937	[114]
PP2A	c-Jun	T239	TLR4	↑	RAW264.7 macrophages	[115, 116]
	IRF3	S396	TLR3, TLR4	↓		[117]
PP4	TBK1	S172			RAW264.7, BMDCs and peritoneal macrophages	[118]
WIP1	p65	S536	TLR4		Splenocytes and astrocytes	[119, 120]

BMMs, Bone Marrow-derived Macrophages; DUSPs, Dual-Specificity Phosphatases; ERK, Extracellular signal-Regulated Kinases; IKK-α, IκB Kinase-α; IKK-β, IκB Kinase-β; IRF3, Interferon Regulatory Factor 3; IRF7, Interferon Regulatory Factor 7; JNK, c-Jun N-terminal Kinase; MEFs, Mouse Embryonic Fibroblasts; PP1, Protein Phosphatase 1; PP2A, Protein Phosphatase 2 A; PP4, Protein Phosphatase 4; PTPN4, Protein Tyrosine Phosphatase, Non-receptor type 4; PTP-PEST, Protein Tyrosine Phosphatase-PEST; RAW264.7, mouse macrophage cell line; S, Serine; T, Threonine; TAK1, Transforming growth factor-β-Activated Kinase 1; TBK1, TANK-Binding Kinase 1; TLR, Toll-Like Receptor; TRAM, TRIF-Related Adaptor Molecule; U-937, human macrophage cell line; WIP1, Wild-type p53-Induced Phosphatase 1; Y, Tyrosine

#### Protein tyrosine phosphatase non-receptor type 22 (PTPN22)

PTPN22 is expressed in lymphoid tissues where it promotes type I IFNs production following TLR3 and TLR4 ligation [83]. Mechanistically, it has been shown that PTPN22 directly interacts with TRAF3, enhancing its K63-linked ubiquitination and consequently IFNs induction. In a model of IL-1β-dependent inflammatory arthritis, *Ptpn22*^*−/−*^ mice develop severe arthritis despite Poly I:C treatment, which usually attenuates disease symptoms [84]. Notably, a single nucleotide polymorphism in *PTPN22* gene, encoding a tryptophan at amino acid 620, has been described as a susceptibility locus for autoimmune diseases and infections [85]. Accordingly, patients with autoimmune Systemic Lupus Erythematosus (SLE) expressed the *PTPN22* R620W variant and this was associated with impaired TLR7 and TLR8-dependent IFN-α induction [86]. These data argue for a protective role of PTPN22 against autoimmune diseases via the regulation of TRAF3-dependent signaling.

#### Src homology region 2 domain-containing phosphatase-1 (SHP-1)

SHP-1 belongs to the SH2-domain-containing family of non-membrane protein tyrosine phosphatases. It has been broadly studied in immune lineages as its expression is abundant in hematopoietic cells [87]. Several studies have reported that SHP-1 differentially modulates NF-κB and IRF3 activation upon TLR3 and TLR4 stimulation [88]. Indeed, in splenocytes, dendritic cells and peritoneal macrophages, SHP-1 inhibits NF-κB-dependent gene induction while it promotes IFN-β production. In fact, SHP-1 directly interacts with IRAK1 which in turn inhibits IRF3 and IRF7 activation through its kinase activity [88, 89]. Surprisingly, this regulatory function of SHP-1 is independent from its phosphatase activity since it is also observed in macrophages expressing the catalytically inactive SHP-1 mutant (C453S, Table 1, section “PTPs”). This interaction between SHP-1 and IRAK1 occurs through a ITIM-like motif found in the kinase domain of IRAK1 [90].

Notably, SHP-1 has been associated with autoinflammatory diseases and infections. Macrophages from autoimmune Multiple Sclerosis (MS) patients exhibit deficient *SHP-1* gene expression in comparison to normal subjects [91]. In line with these observations, *SHP-1* knockdown in normal macrophages increases LPS-mediated NF-κB responses, up to levels observed in macrophages from MS patients [91]. Aside from these observations, treatment of human macrophages with the specific SHP-1 inhibitor Sodium Stibogluconate (SS) [92] reduces LPS-induced production of IL-27 which then inhibits HIV (Human Immunodeficiency Virus) infection in CD4 T cells [93]. These results suggest a pivotal role for SHP-1 in antiviral immunity.

#### Src homology region 2 domain-containing phosphatase-2 (SHP-2)

As SHP-1, SHP-2 belongs to the SH2-domain-containing family of non-membrane protein tyrosine phosphatases. SHP-2 shares 60% of sequence identity with SHP-1 [94] but in contrast to SHP-1, SHP-2 is ubiquitously expressed [95], regulating many different signaling pathways [96]. The first evidence that SHP-2 regulates TLRs signaling were provided by An et al*.* [97] who demonstrated that SHP-2 inhibits IFN production in response to TLR3 and TLR4 ligands. Indeed, SHP-2 deficiency significantly enhanced LPS and poly(I:C)-induced IFN-β luciferase reporter gene expression in macrophages. Interestingly, this function of SHP-2 occurs in a phosphatase activity independent manner [97]. Molecularly, SHP-2 directly interacts with the kinase domain of TBK1, inhibiting IRF3 activation and IFNs production (Fig. 3). These observations were confirmed by Xu et al. who demonstrated that *Shp-2*^*−/−*^ macrophages secrete higher amounts of IFN-β upon TLR3, TLR4 and TLR9 activation in comparison to wild-type macrophages [98]. On the other hand, increased TRAF6 ubiquitination and NF-κB activation were observed in *Shp-2*^*−/−*^ macrophages stimulated with LPS, suggesting that SHP-2 suppresses NF-κB pathway activation in response to LPS.

Regarding the MAPK pathways, it has been reported that conditional *Shp-2* deletion in murine podocytes attenuates JNK and p38 MAPK activation in response to LPS [99]. Similar observations were reported in LPS-stimulated bronchial epithelial cells [100]. However, the molecular mechanisms involved in such regulation remain to be clarified. One could speculate that SHP-2 modulates TRAF6 ubiquitination which in turn modulates TAK1 activity. Additionally, some data suggest that SHP-2 might be involved early following TLR engagement. Indeed, conditional expression of an active SHP-2 mutant in murine endothelial cells blocks LPS-induced barrier disruption and this correlates with increased FAK phosphorylation [101].

#### Protein tyrosine phosphatase-PEST (PTP-PEST)

PTP-PEST is a protein tyrosine phosphatase containing a PEST motif (enrichment in P: Proline, E: Glutamate, S: Serine and T: Threonine) [102] which is associated with proteins with short half-lives [103]. Interestingly, enhanced PTP-PEST expression is observed in macrophages after long term stimulation with LPS, Poly I:C or ODN [104]. This increased expression is associated with decreased induction of proinflammatory cytokines and secretion of IFN-β. Such regulation depends on phosphatase activity of PTP-PEST, since overexpression of a catalytically inactive PTP-PEST mutant abrogates NF-κB and IRFs signaling inhibition [104]. Additional data indicate that PTP-PEST directly interacts with IKK-β and dephosphorylates two tyrosine residues (Table 2) [104]. Much more studies are needed to exactly understand how PTP-PEST modulates NF-κB and IRFs signaling following TLRs ligation.

### MAPK phosphatases (MKPs)

MAPK Phosphatases (MKPs) are dual-specificity phosphatases (DUSPs) (Table 1). They dephosphorylate both threonine and tyrosine residues of MAPKs such as ERK1/2, p38α/β/γ/δ and JNK1/2/3 kinases, hence antagonizing their activation and cellular functions [107].

#### DUal Specificity Phosphatase 1 (DUSP1)

DUSP1 is a nuclear MKP targeting the stress-activated MAPKs p38 and JNK, and which is rapidly up-regulated in response to mitogenic and/or stress stimuli [121]. Interestingly, *Dusp1* KO (Knockout) mice exhibit increased sensitivity to endotoxic shock induced by LPS [122]. Indeed, prolonged JNK and p38 MAPK activation as well as increased TNF-α and IL-6 expression are observed in *Dusp1*^−/−^ cells [122]. Aside from these observations, macrophages pre-treatment with a pharmacological inhibitor of DUSP1, namely triptolide, over-activates JNK and p38 pathways following LPS [106, 107] or the TLR2 agonist peptidoglycan [108] stimulations. Since MAPK activation is necessary for the maximal production of cytokines [123], DUSP1 may act as a pivotal regulator of the innate immune response. In this regard, long-term stimulation of macrophages with peptidoglycan or LPS markedly increases DUSP1 mRNA and protein expression [124].

#### DUal Specificity Phosphatase (DUSP16)

DUSP16 shows greater specificity for JNK and p38, with little or no activity towards ERK1/2 [105]. Notably, macrophages from *Dusp16*^*−/−*^ mice show enhanced JNK activation with an overproduction of IL-12 (Interleukin 12) following LPS and CpG stimulation [109]. As observed for DUSP1, long-time exposure of macrophages with TLR4 or TLR9 ligands promotes *Dusp16* transcription.

#### DUal Specificity Phosphatase (DUSP4)

While DUSP1 and DUSP16 mostly target the stress-activated MAPKs p38 and JNK, DUSP4 inactivates all three MAPKs (p38, JNK and ERK1/2). Interestingly, *Dusp4*-deficient mice exhibit increased susceptibility to *Leishmania mexicana* infection [110], a parasite targeting TLR4 on macrophages [125]. Enhanced release of pro-inflammatory mediators (IL-6, IL-12, TNF-α and Prostaglandin E2) is indeed observed in LPS-stimulated *Dups4*^*−/−*^ macrophages, which is associated with over-activation of MAPKs (Table 2). Additionally, increased arginase-1 expression and activity are observed in *Dusp4*^−/−^ macrophages, resulting in decreased iNOS formation [110]. Mechanistically, arginase-1 hydrolyses arginine (the substrate of iNOS) to ornithine and urea [126]. Notably, arginase-1 inhibition reverses the effect of *Dusp4* deficiency on *Leishmania* growth. Therefore, these studies indicate that DUSP4 protects against *Leishmania* infection mainly by controlling arginase-1 expression and iNOS production.

### Serine/threonine phosphatases (PPPs)

#### Protein phosphatase 1 (PP1)

PP1 phosphatase is involved in many different cellular processes, including TLRs responses [127, 128]. Indeed, PP1 dephosphorylates IKK-α and IKK-β induced by LPS [111]. PP1 negatively regulates TLRs signaling by dephosphorylating TAK1 on serine 412 [112], a residue targeted by Protein Kinase A (PKA) [129]. Mutation of serine 412 on TAK1 prevents PKA-induced degradation of IκB-α and p38 MAPK phosphorylation in RAW264.7 macrophages [129]. PP1 overexpression in macrophages also abrogates NF-κB, MAPKs and proinflammatory cytokine secretion upon TLRs engagement [112]. In addition, other studies have reported that PP1 regulates the IRFs axis as well [113]. In fact, PP1 interacts with and dephosphorylates IRF3 in macrophages, hence abrogating TLR3 response [113]. Recently, IRF7 has also been shown to be dephosphorylated by PP1 (Table 2) [114]. In line with these observations, inhibition of PP1 phosphatase activity enhances IFN-α production and impairs viral replication in human U-937 macrophages infected with Newcastle Disease Virus [114].

#### Protein phosphatase 2A (PP2A)

The ubiquitously expressed serine/threonine phosphatase PP2A accounts for a large fraction of phosphatase activity in eukaryotic cells [130]. Du et al*.* have shown that PP2A negatively regulates IRF3 activation in macrophages and myeloid cells [117]. In vitro assays demonstrate that PP2A directly dephosphorylates IRF3 (Table 2). In mice, the myeloid-specific knockout of *Ppp2a* results in higher mortality in response to LPS challenge and bacterial infection [131]. Notably, increased phosphorylation of MAPKs and NF-κB (IKK-α/β, p65) signaling effectors as well as enhanced secretion of pro-inflammatory cytokines were observed in BMDMs (Bone Marrow-Derived Macrophages) from *Ppp2a*^*−/−*^ mice [132]. Aside from these observations, it has been shown that PP2A dephosphorylates c-Jun, hence inhibiting its proteasomal degradation in response to LPS [115] (Table 2).

Recently, the catalytic PP2A α-subunit was recognized as a novel protective factor for LPS-induced ARDS (Acute Respiratory Distress Syndrome). Indeed, specific ablation of the catalytic subunit of PP2A (*Ppp2ca*) in alveolar macrophages enhances NF-κB and MAPKs activation and aggravates cytokine secretion in response to LPS [133]. Conversely, adoptive transfer of alveolar macrophages with activated PP2A attenuates lung inflammation. Taken together, these results indicate that PP2A tightly regulates the inflammatory responses induced by TLRs, at least TLR3 and TLR4, by limiting the activation of MAPKs and NF-κB pathways.

#### Protein phosphatase 4 (PP4)

Protein Phosphatase 4 (PP4) is a PP2A-like phosphatase [134] regulating many cellular processes including DNA damage responses or the cell cycle [135, 136]. Notably, PP4 physically interacts with the E3 ubiquitin ligase TRAF6 [137]. Silencing PP4 in RAW264.7 macrophages increases NF-κB luciferase activity following TLR4 stimulation [137]. PP4 may also negatively modulate the NF-κB pathway downstream of TLR4, in part by inhibiting TRAF6 ubiquitination in response to LPS. Interestingly, PP4 expression is significantly upregulated in macrophages after long-time treatment with LPS [137]. Recently, PP4 has been shown to suppress IFN I production upon TLR3 and TLR4 stimulations in a phosphatase-dependent manner [118]. Indeed, siRNA-mediated reduction of the expression of the PP4 catalytic subunit in mouse peritoneal macrophages in vivo resulted in increased IFN I expression after viral infection. In addition, it was shown that PP4 direct interaction with the kinase TBK1 leads to dephosphorylation of serine 172 (Table 2) [118] which is located in the TBK1 activation loop, necessary for kinase activity and downstream IRF3 phosphorylation [138]. Thus, PP4 also acts as a potent negative regulator of TLR-mediated antiviral immunity.

#### Wild-type p53-induced phosphatase 1 (WIP1)

WIP1 is a member of the serine/threonine protein phosphatase PP2C family. WIP1 dephosphorylates the NF-κB subunit p65 on serine 536 within its transactivation domain [139] in response to LPS [119] (Table 2). Mice lacking *Wip1* exhibit increased p65 phosphorylation and expression of target genes following LPS injection. Recently, a negative feedback loop between WIP1 and NF-κB in LPS-induced astrocytes was discovered [120]. Primary astrocytes LPS-stimulated increases *Wip1* transcription and WIP1 protein nuclear colocalization with p65. Conversely, *Wip1* silencing in primary astrocytes results in reduced p65 phosphorylation and cytokine transcription following TLR4 activation [120]. Collectively, these results suggest that WIP1 may provide a potent therapeutic target for neuroinflammation, and more generally for chronic inflammatory disorders (Table 3).

**Table 3:** TLR responses in different phosphatase knockout mouse models

	Mouse models	Challenge	Readout	Effects	References
*Ptp1b*^*−/−*^	C57BL/6 Total KO	LPS, zymosan	Serum TNF-α	↑	[77]
		CpG	Lung IFN I		[78]
*Ptpn22*^*−/−*^	C57BL/6 Total KO	LCM Virus	Serum IFN-α/β	↓	[84]
	Arthritis	Poly I:C			
*Ptpn11*^*−/−*^ (Shp-2)	C57BL/6 Podocyte-specific KO	LPS	*Il-1*β, *Il-6* and *Tnf*α levels in kidneys		[99]
*Dusp1*^*−/−*^	C57BL/6 Total KO	LPS	IL-6 and TNF-α levels in serum	↑	[122]
*Dusp4*^*−/−*^		*Leishmania mexicana*	IFN-γ levels in serum	↓	[110]
*Dusp16*^*−/−*^	Radiation chimeras	LPS	IL-6 and IL-12 levels in serum	↑	[109]
*Ppp2ca*^*−/−*^ (Pp2a)	C57BL/6 Myeloid-specific KO	LPS, *E. coli*	IL-6 and TNF-α levels in serum		[131]
		LPS	IFN-β levels in serum		
*Wip1*^*−/−*^	C57BL/6 Total KO	LPS	*Il1-*β and *Il-6* levels in splenocytes		[119]

C57BL/6, C57 Black 6 mouse genetic background; CpG, Cytidine-phosphate-Guanosine oligonucleotides (TLR9 ligand); DUSP, DUal-Specificity Phosphatase; *E. coli*, *Escherichia coli*; IFN-α/β, Interferon α/β; IFN-β, Interferon-β; IFN-γ, Interferon-γ; IFN I, Interferon type I; *Il-1b*, Interleukin-1 β; IL-6, Interleukin-6; IL-12, Interleukin 12; KO, Knockout; LCM Virus, Lymphocytic Choriomeningitis Virus; LPS, Lipopolysaccharides (TLR4 ligand); Poly I:C, Poly-Inosinic:Poly-Cytidylic acid (TLR3 ligand); PP2A, Protein phosphatase 2 a; *Ptp1b*, Protein tyrosine phosphatase 1b; *Ptpn*, Protein tyrosine phosphatase non-receptor type; SHP-2, Src Homology region 2 domain-containing phosphatase-2; TNF-α, Tumor necrosis factor-α; *Wip1*, Wild-type p53-induced phosphatase 1; Zymosan, TLR2 ligand

#### Intestinal alkaline phosphatase (IAP)

IAP is expressed in the brush border of enterocytes where it plays a key role in gut defense [140]. Interestingly, this phosphatase can also be secreted in both the intestinal lumen and bloodstream [141, 142]. In contrast to other phosphatases, IAP does not dephosphorylate proteins. Instead, its reported substrates include bacterial products such as LPS, flagellin and CpG. Hence, IAP plays a crucial role in the regulation of gut microbiota function [143]. For example, by removing phosphates present on the lipid A moiety (which allows LPS to bind TLR4), IAP reduces LPS toxicity [144]. Accordingly, ectopic IAP expression in intestinal epithelial cells and colon cancer cells markedly reduces LPS-induced NF-κB responses [145]. Likewise, oral IAP administration impairs colitis induction in response to Dextran Sulfate Sodium (DSS) in wild-type mice but not in *Tlr4*^*−/−*^ mice [146]. Therefore, IAP protects against colitis by reducing TLR4 pathways activation. Interestingly, the gut expresses three isozymes for IAP: Akp3, Akp5 and Akp6. *Akp3* (coding for IAP3) KO mice exhibit normal basal intestinal MyD88-inflammatory cytokines levels and similar susceptibilities to Gram- *Yersinia pseudotuberculosis* infection when compared to control mice. However, adult *Akp3*^*−/−*^ mice are immune tolerant to low intestinal dose of LPS. Such endotoxin tolerance, acquired post-weaning, may result from higher TLR4 stimulation during development [141]. This suggests IAP’s LPS detoxifying activity is involved in immune education [147].

## Clinical relevance

As TLRs and some adaptors are involved in many human disorders such as atherosclerosis [6], phosphatases regulating TLR signaling may also be implicated. For example, it has been suggested that DUSP1 is athero-protective, as DUSP1 induction is necessary for the anti-inflammatory effects of shear stress in endothelial cells [148]. In addition, Khadir et al*.* identified circulating DUSP1 as a potential biomarker for chronic inflammation in patients with cardiovascular diseases associated with atherosclerosis [149]. Cheng et al*.* have shown that Geniposide, an emerging immunomodulator [150], is anti-inflammatory in part by upregulating *Dusp1* expression in response to LPS [151]. Geniposide reduces atherosclerotic inflammatory injuries in *ApoE*^−/−^ mice which are prone to atherosclerosis [152]. To date, no treatment against rheumatoid arthritis achieved to target its triggering events. However, it has recently been proposed to screen for *PTPN22* gene signatures, in order to predict patient responses to autoimmune rheumatoid arthritis targeted therapies [153]. PTPN22 has also been recommended as a novel rheumatoid arthritis therapeutic target [154], as well as PP2A for neuroinflammatory disorders [155]. Finally, daily administration of Alkaline Phosphatase has been shown to significantly improve patients ulcerative colitis scores, with clinical effects observed within 21 days [156]. Thus, phosphatases may be directly involved in the regulation of human disorders such as atherosclerosis and are currently recognized as pharmacological targets.

## Coronavirus disease-2019 (COVID-19)

Recent data suggest a role of TLR signaling and phosphatases in patient responses to Coronavirus infection. For example, Mizutani et al*.* observed that p38 phosphorylation was significantly elevated in SARS-Coronavirus (SARS-CoV)-infected cells [157]. Inhibition of p38 phosphorylation almost abolished IL-6 and IL-8 induction. These first results are especially promising for targeting phosphatases to treat SARS. In 2011, an evasion strategy for the IBV (Infectious Bronchitis Virus) coronavirus (IBV) was revealed [158]. Indeed, IBV induces DUSP1 expression, correlating with reduced IL-6 and IL-8 secretion. IL-6 and IL-8 releases are part of the cytokine storm, responsible for the Severe Acute Respiratory Syndrome (SARS). COVID-19 is the most recent coronavirus-mediated acute respiratory illness generating severe symptoms. TLR4 has been identified as a potential receptor for the outer protein spike of SARS-CoV-2 [159]. It has been reported that COVID-19 patients upregulate TLR4-mediated signaling, which exacerbates SARS [160]. The more critically ill patients present highly increased S100 calcium-binding protein A9 (S100A9) blood levels, a TLR4-recognized DAMP. In addition, neutralizing autoantibodies against IFN I were detected in patients with life-threatening COVID-19 [161]. Rare putative loss-of-function variants of X-chromosomal TLR7 are associated with impaired type I IFN in young men with severe COVID-19 [162]. Knowing that both TLR4 and TLR7 trigger NF-κB/MAPK activation and antiviral IFN I, and that these pathways may be involved in SARS-related infections, il would be relevant to consider a role for phosphatases in TLRs signaling during COVID-19 infections.

## Conclusion

This review highlights that phosphatases are key players in TLRs signaling. Interestingly, downregulation of most phosphatases markedly decreased or completely abolished LPS tolerance, highlighting the importance of phosphatases in endotoxin tolerization. In this regard, increased expression and activity of PP2A, PTPN22, PTP1B and MKP1 are observed in LPS-tolerized monocytes and macrophages [124]. One could speculate that phosphatases represent attractive targets to control TLRs-induced inflammatory responses. However, unlike kinases, phosphatases are challenging targets against which to develop specific inhibitors or inducers [163]. Their catalytic sites are permissive, rather shallow and greatly polar, making them hard to target. Recent progress in allosteric or oligomerization inhibitors design reveals new chemical tools that will set future therapies [164]. That being said, there are still numerous outstanding questions at the molecular level that remain to be addressed before considering phosphatases as good therapeutic targets to control TLRs functions. For instance, which PTPs dephosphorylate tyrosine phosphorylated TLRs? How exactly TLRs control the activity of regulatory phosphatases to ensure timely proinflammatory responses? Is that modulation cell type-dependent? Further studies are warranted to dissect the kinase-phosphatase network regulating TLRs signaling pathways.

## Data Availability

Not applicable.

## Abbreviations

ApoE^−^^/^^−^ mice: Atherosclerosis-prone apolipoprotein E-deficient mice
BMMs: Bone marrow-derived macrophages
Btk: Bruton’s tyrosine kinase
C: Cysteine
C57BL/6: C57 black 6 mouse genetic background
Cdc25: Cell division cycle 25
CoV: Coronavirus
COVID-19: Coronavirus disease-2019
CpG: Cytidine-phosphate-guanosine oligonucleotides
CREB: C-AMP response element-binding protein
D: Aspartate
DD: Death domain
DAMPs: Damage-associated molecular patterns
DSPs/DUSPs: Dual-specificity phosphatases
DSS: Dextran sulfate sodium
*E. coli*: *Escherichia coli*
EGF: Epidermal growth factor
EGFR: EGF receptor
ERK: Extracellular signal-regulated kinases
FAK: Focal adhesion kinase
FCP1: F-cell production 1
HADs: Holo-acid dehalogenases
HIV: Human immunodeficiency virus
IAP: Intestinal alkaline phosphatase
IBV: Infectious bronchitis virus
IFN-α/β/γ: Interferon α/β/γ
IFN I: Interferon type I
IKK-α/β/γ/ε: IκB kinase-α/β/γ/ε
IL-1β/6/12/27: Interleukin-1β/6/12/27
iNOS: Inducible nitric oxide synthase
IRAK1/4: Interleukin-1 receptor-associated Kinase ¼
IRF3/7: Interferon regulatory factor 3/7
JNK: C-Jun N-terminal kinase
LCM Virus: Lymphocytic choriomeningitis virus
LMW-PTP: Low molecular weight-protein tyrosine phosphatases
LPA: Lysophosphatidic acid
LPS: Lipopolysaccharides
LRRs: Leucine-rich repeats
MAMPs: Microbe-associated molecular patterns
MAPKs: Mitogen-activated protein kinases
MAPKKs: Mitogen-activated protein kinase kinases
MEFs: Mouse embryonic fibroblasts
MKPs: MAPK phosphatases
MS: Multiple sclerosis
MyD88: Myeloid differentiation primary response 88
ODN: Oligodinucleotide
PAMPs: Pathogen-associated molecular patterns
PDH: Pyruvate dehydrogenase phosphatase
PKA: Protein kinase A
Poly I:C: Poly-inosinic:poly-cytidylic acid
PP1/2A2B/2C/4: Protein phosphatase 1/2A/2B/2C/4
PPMs: Metal-dependent protein phosphatase
PRRs: Pattern recognition receptors
PPPs: Phospho-protein phosphatases
pS/T/Y: Phospho-serine/threonine/tyrosine
PTEN: Phosphatase and TENsin homolog
PTPs: Protein tyrosine phosphatases
PTP1B: Protein tyrosine phosphatase 1B
PTPN: Protein tyrosine phosphatase, non-receptor type
PTP-PEST: Protein tyrosine phosphatase-PEST
RIP-1: Receptor-interacting serine/threonine-protein kinase-1
RTPs: Receptor tyrosine phosphatases
S100A9: S100 calcium-binding protein A9
SARS: Severe acute respiratory syndrome
SCP: Small carboxy-terminal domain phosphatase
SHP-1/2: Src homology region 2 domain-containing phosphatase-1/2
SLE: Systemic lupus erythematosus
SNP: Several single nucleotide polymorphism
SS: Sodium stibogluconate
TAK1: Transforming growth factor-β-activated kinase 1
TAB2/3: TGF-β-activated kinase 1-binding 2/3
TANK: TRAF family member-associated NF-κB activator
TBK1: TANK-binding kinase 1
TIR: Toll/interleukin-1 receptor
TIRAP: Toll/Interleukin-1 receptor adapter protein
TLR: Toll-like receptor
TOLLIP: Toll-interacting protein
TRAF3/6: TNF receptor-associated factor 3/6
TRAM: TRIF-related adaptor molecule
TRIF: TIR-domain-containing adapter-inducing interferon-β
WIP1: Wild-type p53-induced phosphatase 1
Y: Tyrosine
